# Randomized prospective study comparing conventional *In Vitro* Fertilization technique to Intravaginal Culture with the INVOCELL™ device for 3 and 5 days

**DOI:** 10.5935/1518-0557.20220042

**Published:** 2023

**Authors:** Lilian de Freitas Aguiar, Gisele dos Santos Pessanha da Cunha, Karoll Andrea Alfonso Torres Cordido, Francisco Augusto Colucci Coelho1, Tânia Maria Ortiga Carvalho

**Affiliations:** 1 Centro de Infertilidade e Medicina Fetal do Norte Fluminense, Campos dos Goytacazes, RJ, Brasil; 2 Instituto de Biofísica Carlos Chagas Filho, Universidade Federal do Rio de Janeiro, UFRJ, RJ, Brasil; 3 Universidade Estadual do Norte Fluminense Darcy Ribeiro, UENF, Campos dos Goytacazes, RJ, Brasil; 4 Faculdade de Medicina de Campos, Campos dos Goytacazes, RJ, Brasil

**Keywords:** intravaginal culture, INVOCell™, *in vitro* fertilization, prolonged culture

## Abstract

**Objective:**

The objective was to analyse and compare the formation and quality of the embryos developed using conventional in vitro fertilization (IVF) and IVC techniques with an INVOCell™ device.

**Methods:**

Two groups were formed, with eight couples in each, one in culture for three days (D3) and another in culture for five days (D5), using intravaginal culture technique with an INVOcell device and a conventional in vitro fertilization technique.

**Results:**

Embryo formation in Group D5 showed 46.7% (IVC) and 40% (IVF) of recovered blastocysts. In the group D5, the conventional IVF, better embryo development dynamics was observed, with 66% of expanded blastocysts, against 28% in the IVC. Group D3 showed 75% (IVC) and 53% (IVF) of embryo formation. Embryonic quality in Group D3 demonstrated that IVF embryos had a better synchrony in the number and quality of blastomeres. All embryos recovered in Group D3, in both techniques, did not show fragmentation. The pH of the medium contained in the INVOCell™ device in both Groups D5 and D3 showed no differences. The means were 7.26 and 7.25, respectively. The pH of the medium used in IVF was 7.29 in both groups. Microbiological analyzes of the culture media contained in the INVOCell™ devices used in Group D5 were negative.

**Conclusions:**

The results showed that the IVC technique, using the INVOCell™ device, provided a healthy and balanced environment for the development and obtaining of quality embryos with three and five days of culture.

## INTRODUCTION

Infertility is a public health problem that according to the World Health Organization (WHO, 2018) affects about 50 to 80 million people of reproductive age (Allahbadia, 2013; Daar & Merali, 2002). In Brazil, this number reaches approximately 8 million (SBRA, 2019). Key international organizations such as the United Nations and the WHO recognize infertility as a disease that deserves medical care (United Nations, Department of Economic and Social Affairs, 2017; Zegers-Hochschild *et al*., 2009). The financial impact of assisted reproduction treatment (ART) is one of the main limitations to couples access, especially in developing countries where there is an important variation in access to ART (Collins, 2001; Tavares *et al*., 2016; DeWeerdt, 2020). Besides the financial impact from the use of new technologies in ART (Armstrong *et al*., 2019), the ART itself and the associated failures contribute to psychological investment and stress that is not always favourable due to uncertainties involved (Vieira & Coelho, 2013).

The intravaginal culture (IVC) technique using the INVOCell ™ device has being offered as a novelty, and has been a topic in clinical discussions, but this technology is not new (Babcock Gilbert & Polotsky, 2019). In 1988, IVC was proposed as a technique offering a more balanced environment and a less costly procedure, increasing access to reproductive care and maintaining embryonic potential without compromising the possibility of pregnancy (Ranoux et al., 1988). The INVOCell™ device uses the physiological conditions found in the vaginal cavity allowing fertilization and early embryonic development. Therefore, IVC reduces daily manipulations in the laboratory, maintains a more balanced environment with absence of light, temperature variation, pH, and O2 tensions - factors that can decrease embryo viability (Bloise et al., 2014).

In Brazil, Coelho et al. (2013) carried out a study comparing the pregnancy rate of patients undergoing the IVC technique using the INVOCell™ device with the intracytoplasmic sperm injection (ICSI) technique. At the time, there were questions regarding the possible interference of the device in the results of ART through possible contamination in the culture environment by vaginal fluids and in its competence in maintaining the balance of the culture medium. These questions were elucidated with negative microbiological analysis, without compromising the results of the ART (Coelho *et al*., 2013). However, there are no comparative reports in the literature proving the integrity and characteristics of the culture environment using the INVOCell™ device for three and five days of culture.

This study aimed to analyse the rate and quality of the embryos developed using the conventional *in vitro* fertilization (IVF) and IVC techniques with the INVOCell™ device, considering the effectiveness of using the device in providing a healthy and balanced environment for the development and quality of embryos after three and five days of culture.

## MATERIALS AND METHODS

### Study scenario

The study was a prospective, randomized balanced (1:1) between two groups, and comparative in a single centre developed during the professional master’s course, held at the Federal University of Rio de Janeiro (UFRJ). This study was conducted in the city of Campos dos Goytacazes, Rio de Janeiro, Brazil. The participants in this study were all patients undergoing clinical marital infertility research who accessed the municipality’s public infertility assistance program.

The study was submitted for evaluation by the National Research Ethics Council through the Ethics Committee 5244. It was approved under the CAAE code 54436116.8.0000.5244.

### Participants

Eligible participants were couples undergoing ART, with indication of tubal and ovulatory factors, as well as infertility factors without apparent cause, who were aged <35 years, with at least six oocytes recovered after ovarian stimulation, and who agreed to sign the informed consent form (ICF). Patients with endocrinopathies, endometriosis, male factor, and those who did not agree with the ICF were excluded.

The study was carried out at the Reproductive Medicine Service of the Hospital Escola Álvaro Alvin/Center for Infertility and Fetal Medicine of Northern Fluminense, located in the city of Campos dos Goytacazes, Rio de Janeiro, Brazil. It occurred in the period between March 2017 and July 2018.

The time of conducting the research was associated with the time of the master’s course, with 8 months defined for training and analysis of the data for each group. The first group was decisive for the sample size. During the total period of this pilot study, 160 cycles of ART were performed, the first group formed was Group D5, with embryonic culture for five days. Nine participants were selected, and of these eight were eligible. After the end of the defined period for Group D5, Group D3, with culture for three days, began. In D3, 13 participants were selected and eight were eligible. Participants who were not allocated to groups, one for Group D5 and five for Group D3 did not reach the minimum number of oocytes needed (6 oocytes) to participate in the study.

**Figure 1:**
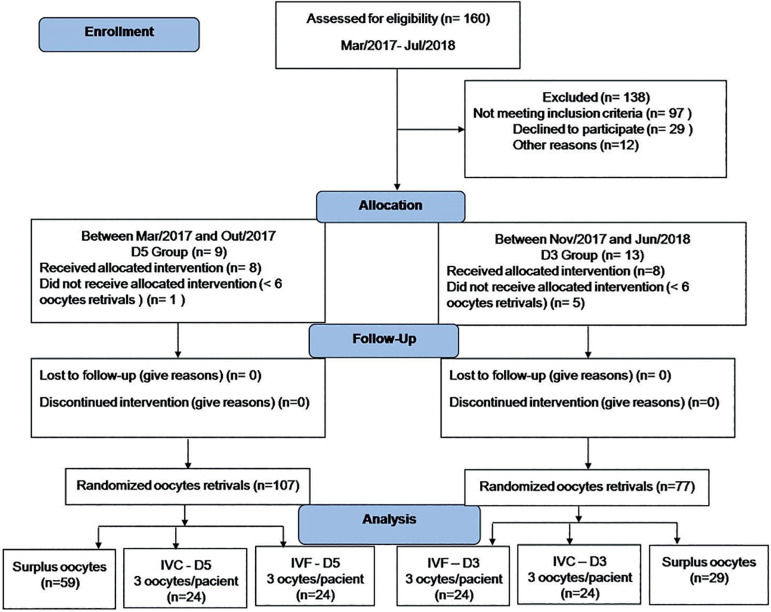
Flow Diagram and follow-up study. 160 cycles of ART were performed, nine participants were selected, and of these eight were eligible and formed Group D5 and thirteen participants were selected and eight were eligible and formed Group D3.

The number of six oocytes was established in order to have a balanced randomization between the evaluated IVC and IVF techniques. Randomization of oocytes was performed by drawing a lot immediately after follicular aspiration, even before oocyte classification. Three oocytes were used in the IVC technique with the INVOCell™ device and three oocytes in the IVF technique in an incubator. Oocytes considered surplus, and those which exceeded the required six, were submitted to the ICSI fertilization technique (Figure 1).

## INTERVENTIONS

### Ovarian stimulation, cycle monitoring, and follicular aspiration

Ovarian stimulation was performed in all participants using 150 µi of urinary gonadotropin until the 10^th^ day or until two follicles reached 16 mm. Indomethacin 50 mg was subsequently administered orally three times a day to block ovulation. The trigger was performed with a GnRH agonist when at least two follicles reached 18 mm. During ovarian stimulation, ultrasound exams were performed every three days to monitor follicular development. Ovarian aspiration occurred 36 h after the trigger.

### Semen preparation

The analysis followed the guidelines of the World Health Organization, 2010 (WHO, 2010). Only samples classified as normospermic were used. The seminal processing was performed by the swim-up method, according to the protocol defined by the Reproductive Medicine Service of the Hospital Escola Álvaro Alvin/Center for Infertility and Fetal Medicine of Northern Fluminense (Volpes *et al.*, 2016). After processing, an aliquot containing a concentration of 40.000 recovered sperm/ml was used, in accordance to the protocol written by Ranoux (2012). The volume used for each fertilization technique is variable according to the recovered sperm concentration (Ranoux, 2012).

### Fertilization by intravaginal culture with the invocell™ device

The cumulus oophorus complex were inseminated in the INVOCell™ device following the manufacturer’s recommendation, which is to use continuous culture medium supplemented with 10 % synthetic serum (Ingamed®) and a concentration of 40.000 sperm recovered/ml, with a volume of 1000 µL. The device was subsequently inserted into the vaginal cavity with the retaining diaphragm to maintain its position. The withdrawal occurred on the 3^rd^ or 5^th^ day after placement, according to the group inserted. The recovered embryos were classified and then cryopreserved.

### Conventional IVF

IVF was performed using the technique of a single well with continuous culture medium supplemented with 10 % synthetic serum (Ingamed®) and a concentration of 40.000 sperm recovered/ml, with a volume of 800 µL and covered with 200 µL of mineral oil. The inseminated cumulus oophorus complex were cultured for five or three days according to the group inserted, without handling of the plate and the incubator, under 7 % CO_2_ at 37ºC. The recovered embryos were classified and then cryopreserved.

### Outcomes

The primary outcome of the study is related to the formation of the embryos, both on the 5^th^ day (Group D5) and on the 3^rd^ day (Group D3). The percentage of formation of embryos, as well as their classification, were the most important parameters to evaluate the effectiveness of the studied techniques.

The embryonic classification of Group D5 was performed according to the degree of blastocoel expansion (1 to 4), 1 - early blastocyst, 2 - blastocyst, 3 - expanded blastocyst and 4 - hatched blastocyst (Alpha Scientists in Reproductive Medicine & ESHRE Special Interest Group Embryology, 2011). The embryos of Group D3 were evaluated for cell count and degree of fragmentation (Alpha Scientists in Reproductive Medicine & ESHRE Special Interest Group Embryology, 2011). For better presentation of the results, the embryos were classified into two scores of 2 to 7 cells or 8 to 12 cells.

To calculate the cleavage rate, the number of degenerate oocytes found after culture was excluded.

The secondary outcome is related to the quality of the culture medium recovered from the INVOCell™ device. The pH and microbiological analysis were the parameters evaluated to prove the safety and efficacy of the device in keeping the culture medium balanced.

The pH measurement was performed in both groups immediately after the recovery of the embryos from the INVOCell™ device, and the IVF plate using a pH meter device (OHAUS starter3100). The ideal pH value of the culture medium was between 7.26 and 7.33. A 100 µl aliquot of the culture media of the participants in Group D5 were subjected to analysis by Total Automation WalkAway (Dade-Behring), identifying fermenting and non-fermenting Gram-Negative bacteria, Gram-Positive Cocci and Listeria, Anaerobic, Yeasts and fastidious microorganisms such as Neisseria and Haemophilus.

### Statistical methods

Data analyses were performed using Student’s *t-*test, Fisher’s exact test, or Chi-square goodness of fit test, where appropriate (Significance level of 5%).

## RESULTS

The flow of study participants can be seen in Figure 1. The profile of the couples included in the study can be seen in Table 1. The average female age was 29.5 in Group D3 and 29.9 in Group D5. Male participants had an average age of 33.1 in Group D3 and 35.6 in Group D5. In the analysed variables, no difference was found between the groups (*p*>0.05). The infertility factors present in both groups were tubal factor and infertility without apparent cause.

**Table 1:** Demographic parameters of the elegible couples.

	D3 (n=8)		D5 (n=8)		*p* value*
Female Age (years)	29.5±1.3 (26.4 - 32.6)		29.9±1.2 (27.0 - 32.7)		0.84
Male Age (years)	33.1±1.9 (28.5 - 37.7)		35.6±2.2 (30.4 - 40.8)		0.41
Infertility Cause	Tubal factor	Idiopathic	Tubal factor	Idiopathic	----
	6	2	5	3	

Values are expressed as mean ± standard deviation and in parentheses the 95 % confidence interval.

* T test (5 % significance).

D3= participants of group with embryonic culture for three days

D5 = participants of group with embryonic culture for five days.

The general results of the study can be seen in Table 2. During the 16 cycles of ART, 184 oocytes were recovered; 96 oocytes were used in the study, divided into 48 for Group D5 and 48 for Group D3. The surplus oocytes were submitted to the ICSI technique. The degenerate oocytes recovered in both groups proved to be inherent factors in the germ cells and not in the fertilization techniques used.

**Table 2:** Comparison of IVC and IVF technique results between the D3 and D5 groups.

	D3				*p* value	D5				*p* value
N° of Retrieval oocytes	Total 77	IVC 24	IVF 24	Surplus 29	----	Total 107	IVC 24	IVF 24	Surplus 59	----
	**IVC**		**IVF**			**IVC**		IVF		
N° of Retrieval oocytes post culture	Degenerate: 4 Cleaved: 15 Not Fertilized: 5		Degenerate: 5 Cleaved: 10 Not Fertilized: 9			Degenerate: 5 Cleaved: 15 Not Fertilized: 4		Degenerate: 6 Cleaved: 15 Not Fertilized: 3		
Cleavage rate (%) CI 95 %*	75 (15/20) 53-89		53 (10/19) 32-73		0.2	79 (15/19) 56-92		83 (15/18) 60-95		1.00
Blastocyst formation rate (%) CI95 %*	----		----		----	46.7 (7/15) 25-70		40.0 (6/15) 20-64		1.00
Blocked embryo rate (%) CI 95 %*	----		----		----	53.3 (8/15) 30-75		60.0 (9/15) 36-80		1.00

* The 95 % confidence intervals were computed by modified Wald method. Cleavage rate (%) = Total of cleaved embryos/Total retrieval oocytes post culture, except degenerate. Blastocyst formation rate (%) = Total of formed blastocysts/Total of cleaved embryos. Blocked embryo rate (%) = Total embryos that did not reach blastocyst/Total of cleaved embryos. IVC = Intravaginal Culture, IVF = In vitro Fertilization, D3= embryonic culture for three days, D5 = embryonic culture for five days.

One limitation of our study was the lack of evaluation of the fertilization rates due to the unavailability of the device and the need to maintain the incubator environment using the IVF technique. The incubator used for the IVF group remained unopened to prevent interference such as exposure to light and variations in temperature, which could interfere with the results.

There was no significant difference for the cleavage rate, in Group D5 79% (IVC) and 83% (IVF); in Group D3 75% (IVC) and 53% (IVF). In the blastocyst formation rate, Group D5 presented 46.7% (IVC) and 40% (IVF). The embryos blocked in Group D5 were 53.3% and 60% for IVC and IVF, respectively (Table 2).

**Figure 2:**
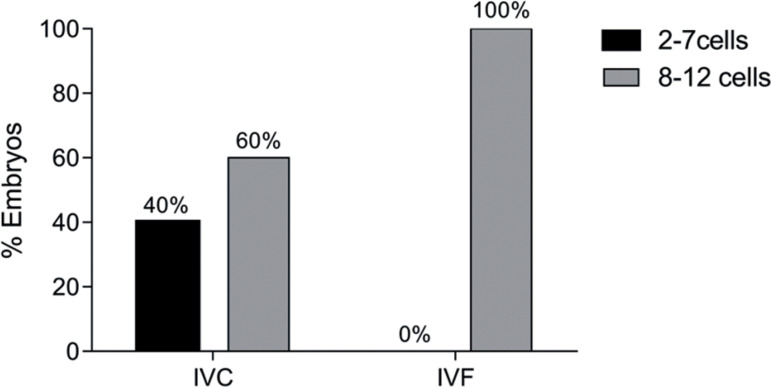
Embryonic classification of the D3 Group in two scores, 2 to 7 cells (Black) or 8 to 12 cells (Gray). Embryos IVC culture (n=15), embryos IVF culture (n=10). IVC = Intravaginal Culture, IVF = *In vitro* Fertilization. (Fisher’s exact test: *p*-value>.0001).

The analysis of the quality of the embryos recovered in Group D3 showed that the embryos from IVF showed a better synchrony in the number and quality of the blastomeres (*p*<0.0001), 100% of which were in the score 8-12 blastomeres. Of those from IVC, 60% were in the score 8-12 blastomeres and 40% in the score 2-7 blastomeres (Figure 2). None of the embryos recovered in either technique showed fragmentation.

**Figure 3:**
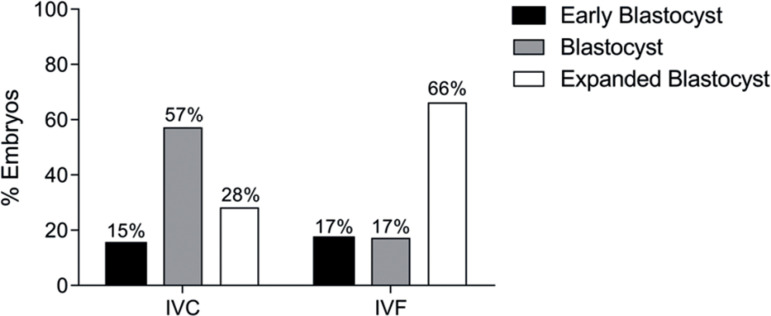
Blastocyst classification according to degree of expansion of the blastocele, of the D5 Group. Embryos IVC culture (n=7), embryos IVF culture (n=6), IVC = Intravaginal Culture, IVF = *In vitro* Fertilization. (The Chi-Square test: *p*-value<.0001).

When comparing the results found in Group D5 in both techniques, based on the cleavage rate, the result shows an equivalence in the formation of a blastocyst (*p*>0.05), with 46.7% in the IVC and 40% in the conventional IVF (Table 2). The blastocysts recovered from the conventional IVF group showed better embryonic development dynamics (*p*<.0001), with 66% of expanded blastocysts, compared to 28% in the IVC group (Figure 3).

The measurement of the pH of the medium contained in the INVOCell™ device in both Groups D5 and D3 showed no differences (D5 7.26±0.05 and D3 7.25±0.07, *p*>0.05). The culture medium used in the IVF technique had a pH of 7.29±0.006. The microbiological analyses of the culture media of the INVOCell™ devices used in Group D5 showed a negative result for the microbiological agents evaluated.

## DISCUSSION

Intravaginal culture using a gas-permeable device (INVOCell™) has been used as a proposal for a balanced environment for embryonic culture and with more accessible costs. By lowering costs, we were able to make assisted reproduction more democratic, reaching more couples with reproductive challenges. Studies carried out in several countries have shown good results using the technique in a three day culture (Coelho *et al.,* 2013; García-Ferreyra *et al.*, 2015; Lucena *et al.*, 2012; Mitri *et al*., 2015). The use of the IVC technique in prolonged culture until the 5^th^ day of the embryo was reported by Doody *et al.* (2016) with promising results.

In conventional cultures using traditional incubators, embryo development assessments are a well-established lab routine, unlike IVC culture which limits us to post-culture assessments. However, the INVOCell™ device offers a balanced and close to natural environment, maintaining temperature, pH, and O_2_ concentration at physiological levels. Wale & Gardner (2016) reported in their review, that the effects of oxygen concentration on the cleavage rate have been studied since 1970 by Edwards and collaborators. They also showed that when exposed to 5% oxygen concentrations compared to 20% exposure, embryos were of better quality. In the last five years, incubators associated with the time lapse system have been able to associate the O_2_ tension at 5% and the observation of the morphokinetic pattern of the cultured embryos. However, the results of the observations showed no signif-cant difference in the morphokinetic pattern in euploid and aneuploid embryos (ESHRE Working group on Time-lapse technology, 2020; Armstrong *et al*., 2019); thus, demonstrating that there is no evidence that the lack of daily observation when using the INVOCell™ device has a negative impact on the process.

Another factor that impacts embryonic quality are the reactive oxygen species, increased in exposure to large O_2_ tensions and associated with mitochondrial dysfunction, interfering with DNA methylation (Li *et al*., 2016), a factor overcome by the decrease in O_2_ tension in the device.

Some degree of presence of fragments in early-stage human embryos cultured *in vitro* is common. These anuclear cytoplasmic structures are the result of unestablished cellular processes. Their presence is correlated with reduced rates of embryonic implantation. In our study all recovered embryos showed degree 1 of fragmentation, including embryos (40% of IVC) in the score of 2-7 cells.

Several studies have evaluated the etiology of oocyte degeneration. Rubino *et al*. (2016) associated degeneration with ovarian stimulation and excessive manipulation of oocytes by the ICSI technique. Lim & Tsakok (1997) associated the increase in the rate of oocyte degeneration with the age of the woman, and suggested the probability that organelle disorganization induces chromosomal errors in oocyte meiosis, enabling degeneration. However, there is still no definite factor of the cause of the degeneration (Rosen *et al.*, 2006). In our study, the number of degenerated oocytes observed in both groups (IVC and IVF) was similar, which indicates that oocyte degeneration was related to intrinsic factors of the gametes. In our case, age-related degenerated oocytes could not be an issue since the participants were <35 years old and the oocytes manipulation was brief. Prolonged embryo culture is increasingly being used in the routine of assisted reproduction laboratories, helping in embryo selection. For this, a culture system must ofer an adequate environment supplying the metabolic demand of the embryo (Moreno *et al*., 1999). According to Balaban *et al*. (2000) in a retrospective analysis, embryo transfer on the 5^th^ day is associated with an increase in the rate of implantation, although there is only evidence of moderate quality in the increase in the clinical pregnancy rate (Glujovsky *et al*., 2016).

In 2019, Storr *et al*. (2019) clarified the main predictive parameters for blastocyst selection, with the degree of expansion of the blastocoel being considered one of the most important. Based on this observation, the results found in Group D5 show that the culture system meets the embryonic needs in this period. Since 85% and 72% of the blastocysts recovered from IVC and IVF, respectively had a blastocyst and expanded blastocyst classification, the blastocysts recovered from the IVF culture had a greater degree of blastocoel expansion compared to the IVC (Figure 3). The heterogeneity shown in the quality of the blastocysts formed in the device can be attributed to intrinsic factors of the gametes, since the variables pH, temperature, and oxygen concentration were constant in the IVC culture.

It is known that the human embryo has mechanisms to regulate the internal pH (Phillips *et al*., 2000), but a variation in the pH of the culture medium has detrimental effects on the initial embryonic development (Swain *et al*., 2016). The pH values found in the culture medium of the two techniques used, 7.26 and 7.25 (IVC) and 7.29 (IVF) remained within the range suggested by the manufacturer (7.26 - 7.33). The percentage of carbon dioxide and the frequent manipulation of embryos, as well as the opening of incubator doors are important factors that might alter the media pH. These factors were overcome in the IVC technique (Wale & Gardner, 2016).

The microbiological contamination in the use of the IVC technique was one of the limitations in the beginning of its development. However, this was overcome with the use of the INVOCell™ device. In 2013, Coelho *et al*. (2013) proved the safety regarding microbiological contamination by using the device in culture for three days. This study corroborates the safety of using the device in culture for five days.

The use of IVC culture proves to be a viable and safe technique for ART, presenting results equivalent to conventional IVF in culture for three and five days. Even though there is no daily monitoring of embryonic development, the stable environment offered by IVC culture overrides this limitation. The IVC technique with the INVOCell™ device does not replace the IVF technique. However, in some well-selected cases it is a less complex and less expensive alternative that does not compromise the treatment and results when compared to more complex culture systems that ofer the same stable environment, such as bench incubators and incubators with a time lapse system, which present a high cost to laboratories and patients.

The results found proved that the IVC technique with the use of the INVOCell™ device provided a healthy and balanced environment for the development and obtaining of quality embryos with three and five days of culture being a viable and safe option for use in ART laboratories. However, further studies evaluating the content of the culture medium recovered after culture should be carried out. Thus, it is possible to measure the values of the metabolites produced by the embryos, which are essential to assess and associate the observed embryonic quality. A direct analysis of IVC *versus* IVF culture costs in diferent countries and lab realities is also necessary.
